# Innovative communication approaches for initializing pediatric palliative care: perspectives of family caregivers and treating specialists

**DOI:** 10.1186/s12904-023-01269-3

**Published:** 2023-10-10

**Authors:** Lucie Hrdlickova, Kristyna Polakova, Martin Loucka

**Affiliations:** 1 Department of Pediatric Hematology and Oncology, University Hospital Motol, V Uvalu 84, Prague, 5,150 06 Czech Republic; 2 Pediatric Supportive Care Team, University Hospital Motol, Prague, Czech Republic; 3 First Faculty of Medicine, Charles University, Prague, Czech Republic; 4 Center for Palliative Care, Prague, Czech Republic; 5 Third Faculty of Medicine, Charles University, Prague, Czech Republic

**Keywords:** Communication, Pediatric palliative care, Initial consultation, Parental feedback

## Abstract

**Background:**

Effective cooperation between a pediatric palliative care team (PPCT), primary treating specialists, patients and families is crucial for high quality care of children with complex life-limiting conditions. Several barriers among patients, families and treating specialists have been identified in the context of initializing pediatric palliative care. The aim of the study was to assess the experience with initial pediatric palliative care consultations from perspectives of family caregivers and treating physicians with a special focus on two innovative approaches: attendance of the treating specialist and the opportunity for parents to give feedback on the written report from the consultation.

**Methods:**

This was a qualitative study using semi-structured interviews with family caregivers of children with malignant and non-malignant disease and their treating specialists. Framework analysis was used to guide the data collection and data analysis.

**Results:**

In total, 12 family caregivers and 17 treating specialists were interviewed. Four main thematic categories were identified: (1) expectations, (2) content and evaluation, (3) respect and support from the team and (4) consultation outcomes. Parents viewed the consultation as a unique opportunity to discuss difficult topics. They perceived the attendance of the treating specialist at the initial consultation as very important for facilitating communication. Treating specialists valued the possibility to learn more about psychosocial issues of the child and the family while attending the initial palliative care consultation. All participants perceived the written report from the consultation as useful for further medical decisions. Family members appreciated the chance to give feedback on the consultation report.

**Conclusions:**

Our study identified several clinically relevant issues that can help initialize pediatric palliative care and establish effective collaboration between families and PPCT and treating specialists. Supporting treating specialists in their ability to explain the role of palliative care is important in order to reduce the risk of misunderstanding or unrealistic expectations. Developing more specific expectations seems to be one of the ways to further increase the effectiveness of initial consultations. The results of the study can be especially helpful for the initial phase of implementing pediatric palliative care and initializing the process of setting up a collaborative relationship with palliative care teams in the hospital.

**Supplementary Information:**

The online version contains supplementary material available at 10.1186/s12904-023-01269-3.

## Introduction

Over the past decades, specialized pediatric palliative care (PPC) has been developing around the world in order to support children with life limiting conditions (LLC) and their families [1]. Despite robust evidence that palliative care is beneficial for children with LLC and increases their quality of life [2–6], initiating palliative care at the right time and in the right way remains a challenging issue for clinicians [7–11]. Several tools and recommendations have been developed to enable early identification of pediatric patients in need of palliative care [12–14]. Yet, many physicians do not refer children to palliative care or the referral comes late in the disease trajectory [15–18].

The initial consultation with the pediatric palliative care team (PPCT) in hospital setting represents a unique opportunity for the introduction of PPC to the family [19, 20] and often becomes the starting point of advance care planning discussions [19, 21, 22]. However, parents often have preconceived notions about palliative care and what to expect during the initial consultation, which may result in concerns or reluctance to meet [23]. Several misperceptions have also been identified among health care providers and referring pediatricians [8, 24, 25]. Preconceived ideas about palliative care and what will occur in the initial consultation have been cited by families and clinicians as barriers to the referral [24, 26].

In the Czech Republic, PPC in hospital setting was introduced in 2017 by founding the multidisciplinary PPCT in University Hospital Motol, the largest tertiary hospital in the country with 570 beds for pediatric patients. To facilitate the implementation of PPC in the Motol hospital and to increase the education about PPC among health care professionals, treating specialists are invited to attend the initial consultation with the PPCT. Presence of a treating specialist at the initial PPCT consultation enables opened communication and the invitation for the treating specialist has been set up as a standard procedure for the initial family meetings with the PPCT team in Motol hospital.

The primary aim of this presented study was to assess the initial experience with the PPCT from the perspectives of family caregivers and treating specialists, who are invited to attend the initial consultation with the PPCT. Apart from exploring the general experience with the initial consultation, this study also looked at a specific novel approach in documenting the initial consultation, which is used by the PPCT. Parents have the opportunity to read the report and to provide feedback or suggestions for amendments before its upload into the electronic medical record. The purpose of this procedure is to make parents more involved and in charge of how the story of their family is described in medical records.

## Methods

To answer the research questions, a qualitative study using semi-structured interviews and framework analysis was conducted [27]. The study is reported following the Consolidated Criteria for Reporting Qualitative Research (COREQ) [28]. (Supplementary 1, online).

### Sample

A purposeful sample of Czech-speaking parents of children with LLC referred to the PPCT and of treating specialists was recruited. Parents were sampled based on the diagnosis, age and illness phase of the child to achieve maximum variety of the sample. All parents had experienced initial palliative care consultation, which traditionally takes place in a family room of the PPCT. It is a cosy comfortable room with a sofa, armchairs and paintings on the wall with no obvious medical instruments or equipment. Primary treating specialists were invited to the study if they had participated in the initial consultation with the PPCT. All participants were recruited via email sent by the member of the PPCT.

### Data collection

Semi-structured interviews were conducted between January 2021 and June 2021 by two palliative care nurses and two paediatric oncologists supervised by the leading author. To minimalize bias, interviewers were assigned to the family caregivers with whom they did not have previous acquaintance. Written informed consent was obtained from all participants.

Semistructured interview guides with a set of open-ended questions were used (Supplementary 2, online). Guides were piloted with four parents and two treating specialists to test its feasibility. Parents were interviewed via telephone or in person, physicians only in person. The face-to face interviews took place at the PPCT´s family room. The interviews lasted from 20 to 90 min. Field notes were made after each interview. The interviews were audio recorded, anonymised and transcribed verbatim in Czech. For the purpose of this article, participants´ quotes were translated into English using an online translator with the translation being checked and finalized by the main author.

### Data analysis

Data was analysed by two researchers (LH, KP) using framework analysis [27], a research method reccommended for qualitative research in medicine [29]. Audio recordings were listened to and transcripts read in full. Contextual and reflective notes were taken during this process. After familiarization, four purposefully chosen transcripts were read and coded by the open coding method. After this process, the researchers discussed their interpretations and coding of the data and initial coding framework of 50 codes was developed. Codes were grouped into 10 categories using a tree diagram and a working analytical framework was formed. The framework was used for indexing the remaining transcripts with four more codes emerging during this process. The final code book used for coding all of the interviews consisted of 10 categories with 41 codes in total (Supplementary 3, online).

In the next phase, all coded data was charted into the framework matrix by using a spreadsheet in Excel. Non-interview data such as field notes and reflexive considerations was added to the matrix. Via this process codes and categories were transformed into themes. Saturation was reached on a conceptual level.

## Results

In total, 22 family caregivers were invited to the study. The study included 12 family caregivers (seven mothers, four fathers and one grandmother) of 10 children including three bereaved family caregivers of two deceased children. Response rate was 54%. Median interval between the initial consultation and the interview was six months with the minimum of 3-month interval and maximum 24 months from the initial meeting. Parents refused participation because of lack of time (n = 6) or for unknown reasons (n = 4). Characteristics of family caregivers and pediatric patients are presented in Table 1.

**Table 1:** Characteristics of the family caregivers (N = 12), their children (N = 10) and treating specialists (N = 17)

Characteristic	Number (N)	Percentage (%)
Family caregivers		
Gender		
Male	4	33
Female	8	66
Age (years)		
30–40	6	50
over 40	5	42
Unknown	1	8
Marital stage		
married/cohabiting	9	75
divorced/not cohabiting	3	25
Interval between the initial consultation and interview with the researcher		
3–6 months	5	42
7–12 months	5	42
13–18 months	1	8
19–24 months	1	8
Pediatric patients		
Age of the child (at the interview) (years)		
0–1	4	40
2–5	2	20
6–12	3	30
over 12	1	10
Gender of the child		
Male	6	60
Female	4	40
Diagnoses		
Non-malignant disease (total)	5	50
Epileptic encephalopathy	1	10
Congenital anomalies	3	30
Cardiovascular disorder	1	10
Malignant disease (total)	5	50
Bone/soft tissue sarcoma	2	20
Neuroblastoma	2	20
Leukemia	1	10
Subspecialty of treating specialists		
Pediatric oncology	14	82
Pediatric cardiology	1	6
Pediatric nephrology	1	6
Pediatric neurology	1	6

Out of 20 invited primary treating specialists, 17 agreed with participation (85% response rate). All physicians were specialized in pediatrics with the following subspecialty: oncology [14], cardiology [1], nephrology [1], neurology [1].

## Findings

The following four main themes were identified: [1] expectations, [2] content and evaluation, [3] respect and support from the team, and [4] consultation outcomes (Supplementary 4, online). Quotes in the following text refer to family caregivers (F) and treating specialists (TS).

### Expectations

Expectations from the initial palliative care consultation differed between family caregivers and health care providers. Family caregivers reported insufficient knowledge about the PPCT and palliative care in general, which resulted in “no expectations” prior to the consultation. Several parents expected an improvement of communication with the primary treating physician. Some family caregivers were worried about upcoming difficult discussions which they expected to happen during the consultation.

*I had no expectations, I hoped that by dealing with the palliative and support team, the situation would improve, that the communication between us and the doctors would improve.* (F7)

*Actually, I knew that things were already looking bad with my son. Because, the word palliative, I know what it means, that kind of accompaniment on the last journey, that’s what I expected from it.* (F12)

### Content and evaluation

Both family caregivers and primary treating specialists highly valued the content of the initial palliative care consultation. Three main topics were identified: 2.1 unique communication, 2.2 practical aspects of the consultation, 2.3 specific topics discussed.

#### Unique communication

Family caregivers considered the initial palliative care consultation to be different from other consultations they had experienced. They appreciated honest and truthful communication and valued the PPCT members´ empathy during difficult discussions. All participants positively acknowledged several aspects of communication. Enough time enabled the attending persons to open difficult topics, while active listening encouraged family members to voice their hopes and concerns. Parents felt like “being heard”, not only by the members of the PPCT but also by the treating specialist who attended the PPCT consultation.

*… through this I feel there is a lot of honesty. And that seems important to me, when a person asks something directly and wants to know the answer, he gets it directly. Even if the answer should be some kind of horrible or the answer should be “we really don’t know”. So I appreciate that and I consider that the most important thing.* (F05)

*So we were basically very happy that someone paid attention to us, that someone gave us a lot of time.* (F11)

Both time and listening facilitated communication with the treating specialist and therefore with the whole primary treating team. Parents also highly valued the information structure, which helped to navigate them through the consultation and gave them space to absorb new information. They also appreciated that members of the PPCT took away the communication burden by asking the primary physician difficult questions thus acting as an advocate of the family.

*And thanks to the fact that the [palliative care] doctor asked the questions that the [primary] doctor and I hadn’t said to each other before and that were kind of in the background, everything became clear and we openly talked about it, and the primary treating specialist was glad that we understood it.* (F07)

Although parents often found the content of the consultation to be upsetting, they remember receiving serious news as an exceptional and valuable moment for the future.

*I was completely excited about it. Of course it was a very heavy and sad subject and all, but I felt good about it. I was certainly satisfied − 100%.* (F12)

Primary treating specialists valued the initial palliative care consultation especially because they gained **new information** regarding parents´ preferences, wishes, concerns and uncertainties and about the complexity of the family situation.

*It is very much focused on the perspective of the family itself, what they wish for, how they would like to arrange it, what they possibly want, what the child himself wants.* (MD13)

#### Practical aspects of the consultation

The number of people attending the initial consultation was identified as an important practical aspect. Family caregivers highly evaluated attendance of various members of the team and also the presence of the primary treating physician. Treating specialists reported concerns about numerous members of the team being present. Parents shared this concern, too. All participants appreciated the PPCT family room as a pleasant place, very different from where difficult discussions usually took place in the hospital.

*At the first session, it was hard to capture the number of participants and who was who, but immediately it was obvious, who was more needed and who was less needed. They also responded to what we said, so there was always a specific person who could help with something.* (F09)

*Until now, they were used to one, two or three doctors talking to them, suddenly they find themselves in front of a committee and they can have different feelings, different stresses…* (MD01).

#### Specific topics discussed

Future care possibilities and a treatment plan represented the crucial topics discussed during the initial palliative care consultation. All participants valued the opportunity to talk about illness-related topics which had not been discussed before, such as possible scenarios, likelihood of cure or the risk of life-threatening complications.

*At that first consultation, the most important thing was that they actually negotiated with us that he doesn’t have to have a feeding tube, that he doesn’t have to live with a ventilator, … that we can go home without any devices and that they take my wishes into account and that, well, let us do it. So I perceived it as a huge win…* (F06).

*The entire course of that consultation is certainly important for opening up the sometimes problematic questions at the end of life and what they actually wish for, what is their idea of ​​the scenario of the last days* (MD10).

Psychosocial aspects discussed during the consultation included psychological well-being of the patient, the parents and siblings, social background of the family and psychosocial needs. Primary treating specialists perceived the consultation as a unique opportunity to gain information about psychosocial needs of the patient and their family.

*… various detailed information is given there about the feelings of the family that they are experiencing, about the fact that there is space for the parents to express their wishes, about what we can do for them, … how they perceive it and some future fears of their siblings and all those affected by the situation.* (MD03)

### Respect and support from the team

This theme is related to mutual respect and support and includes 3.1 parental role and 3.2 long-term support.

#### Parental role

All participants perceived the parental role as being a partner for the primary treating physician in discussing not only questions about treatment related topics, but also for communicating the parents´ and child´s wishes. Respectful attitude of the PPCT members encouraged parents to truly act as partners in the debate. Parents felt encouraged by the PPCT members to express their worries, hopes and concerns. They highly valued being supported in their **parental autonomy**. This approach enabled them to voice their preferences for the first time, sometime about very important clinical issues including stopping of chemotherapy, preferring comfort care or staying at home rather than prolonging life.

*… basically we got a space, a big space to express ourselves and ask the things we needed or wanted to ask.* (F11)

#### Long-term support

Both parents and treating specialists perceived easily accessible long-term support from the PPCT as an important type of care for managing difficult situations in the future, especially for patients discharged from the hospital to home care.

*Thanks to the consultation, they will understand that they will need palliative care, they suddenly understand that these are the people in their place and that it is not some administratively important additional care, but that it is really the care that they will need now.* (MD01)

## Consultation outcomes

Two main topics were identified regarding the consultation outcomes: 4.1 written report and 4.2 practical information obtained.

### Written report from the initial palliative care consultation

Parents highly valued the written report from the consultation, especially because the content of the conversation remained and could not be falsely interpreted. Also, the document from the consultation served as an important information source. Family caregivers also found important that their wishes and preferences were reported for primary care teams for further treatment decisions via an official note from the palliative care consultation.

*So this is like very helpful in that I feel like the message is really accurate, that it matches what’s going on.* (F03)

Treating specialists described the content of the written report as covering many areas which are not usually covered in medical records, specifically appreciating the information about psychosocial aspects, parental opinions and preferences, and the “personal style” of the report. They also emphasised the importance of a written report as a prevention of future conflict and for a potential legal use.

*The report from the palliative care team clearly covers all the information about the family and, above all, what needs to be done for the child and the family… in this it brings us a lot of information and for us it is an important source in how then continue to work and communicate with that family.* (MD01)

The opportunity for **authorisation** prior to making the report from the consultation an official part of the medical record was very much appreciated by both the parents and the treating specialists.

*We read it and I say, well, this sentence may sound like we are giving-up here because of this, and we didn’t mean it that way. And now we remembered that, the doctor said that we can change it and that’s good.* (F09)

*Parents normally don’t have a say in the medical documentation and if they can like this, then I think this is a step that will encourage lot of confidence in them.* (MD05)

Although treating specialists appreciated the possibility for the report being authorised by parents, they were concerned the authorisation may result in reducing information in the report. On the contrary, family members attending the consultation were grateful for the opportunity to remove topics which they would find highly private, too emotional or controversial, and shorten the written report from the consultation prior to its filing in the electronic hospital system.

*It’s a statement, actually like not exact information or a lot of personal things, but which can be read by anyone who looks into the documentation, so it’s certainly fair that the parents have access to it, that they can see it. Because then it can be read by nurses, doctors, the team that has access to it is like a wider one, so that’s definitely good.* (MD03)

The reasons for high appreciation of the written report and the possibility to authorise it are summarized in Table 2.

**Table 2:** Feedback on the written report from the initial palliative consultation from family caregivers and primary treating specialists

Reasons for high validation of the written report from the initial consultation reported by family caregivers and the treating specialists
Family caregivers
• content of the conversation is maintained
• parental preferences are available for primary care teams for further treatment decisions
• notes serve as an information source for all
• notes are available for future reading
Treating specialists
• specialist gain insight in the topics discussed if they missed the consultation
• official report including parental preferences strengthens the relationship with the PPCT
• useful from the legal point of view, prevention of conflict
Reasons for usefulness of the opportunity for family caregivers to give feedback on the report from the initial consultation reported by family caregivers and treating specialists
• expectations of the family and the PPCT regarding the written report from the consultation is harmonized
• understanding parental preferences is verified before being inserted to the official documentation
• erasing controversial or highly personal information is possible when parents wish to do so
• potential misunderstanding and misinterpretation of parental preferences is minimalized
• supporting the shared-decision process with parents acting as partners in the debate
• gesture of trust

### Practical information obtained

Palliative care consultation outcomes were represented also by a set of practical information delivered to the family. Parents received information about the forms of support offered by the PPCT and how the care would be provided. Parents appreciated having **contacts of PPCT members, including phone numbers and emails**. Primary treating specialists positively acknowledged that precise information about palliative care and the work of the PPCT was introduced. According to the primary specialists, that was when parents realized the true value of **multidisciplinary team work**.

*Because they explained to us why we came under the care of the palliative care team, what the team does, what it can do in the future, but at the same time I learned that we may not need their service at all.* (F05)

*Thanks to the consultation, the parents will understand that they will need palliative care, that these are the people in their place and that it is not some additional care, but that it is really the care that they will need now.* (MD01)

## Discussion

Our results showed that the initial consultation with the PPCT is well accepted by both parents and treating specialists, who appreciate supportive communication, empathy, time and practical help that is provided to families during the consultation. However, several challenges need to be acknowledged to maximize the benefits of the consultation.

Previous research suggests that parental expectations from a palliative care consultation play an important role [21, 23, 30]. The study by Daxer et al [21] showed that profound differences between the expectations of parents and health care providers led to misunderstanding and discontent at the initiation of palliative care. In Monterosso´s study, parents perceived palliative care as the “beginning of the end” and expressed uncertainty regarding the transition process to palliative care(30). As Verberne´s article reveals, parents perceived clarifying of PPCT value and content of PPCT support in advance as the main area for improvement(23). Interestingly, in our study, parents reported either “no expectation”, or being somewhat concerned. Parents were usually informed about palliative care by the treating physician, which could impact their expectations, as previous research suggests that physicians and other health care providers lack sufficient knowledge about palliative care [25, 30–32].

Our study revealed that the attendance of the treating physician at the initial palliative care consultation was highly valued by both the parents and physicians. Several studies reported data regarding the practical aspects of the palliative care consultation [19, 20, 22], yet little data is available on who should participate. The review by Bradford et al [20] identified that mostly primary treating physicians participated at the consultation, and suggested other specialists´ attendance should be considered. To our knowledge, no recommendation towards the attendance of the treating specialist at the initial palliative care consultation has been published. Our findings suggest that the attendance of the treating physician may improve physicians´ knowledge of the concept of palliative care. It may also increase their readiness to initiate advance care planning, which pediatricians feel to be unprepared for [33]. The treating physician attending the initial PPC consultation may also represent an important step towards advocating for palliative care in the hospital and to improve implementation of PPC, which can be very challenging [7, 8, 10].

One of the striking findings in our results was the paramount focus on the value of embracing parents´ autonomy and respecting parental preferences reported by all participants. Respectful attitude identified in this study is helpful in building trust and establishing rapport with the family which is consistent with previous research [20, 22, 23, 30]. Similarly to Daxer´s study [21], our study reveals that enough space and active listening transformed the initial palliative care consultation into a strong unique experience for the parents. Furthermore, our findings showed that treating specialists highly valued respectful approach and supporting parents in their parental role. This was a surprising finding, as in the Czech Republic, traditional paternalistic approach is applied in most medical situations [34]. Rapoport´s article on Czech versus Canadian culture differences in providing pediatric palliative care suggests that Czech physicians tend to be more directive than physicians in North America [35]. As previously published, titrating the appropriate level of directiveness with patients and families of children with LLC may be very challenging [30, 36]. However, there is a lack of published data on parental preferences or physicians´ perspectives regarding directiveness from the region of Central and Eastern Europe.

A written report from the palliative care consultation represents a key outcome in the context of providing patient- and family-centred palliative care [22]. Insufficient documentation can jeopardise the provision of quality health care; regardless of whether the discussion did not take place or the notes from the discussions were not taken [22]. As Zhukovsky [37] showed, only documented communication makes possible to evaluate if interventions are tailored according to child´s and family´s goals of care. Both parents and treating specialists found that the authorisation of the report before it is filled in the medical record was an important opportunity to truly engage parents in decision making. Furthermore, possibility of authorisation was perceived as a unique way to gain parents´ trust, which stands for the key aspect of clinician - family relationship in pediatrics [38].

There are several limitations of the study. Firstly, our findings are based on data from one hospital, thus limiting the transfer of the findings to different settings. Secondly, participating physicians were dominantly pediatric oncologists thus the study offers minor insight regarding perception of pediatricians with other subspecialties. The study lacks the insight of other health care providers beside physicians. Other limitation is regarding the data collection which was done retrospectively, thus possibly affecting participants´ recollection of their experience. Also, no comparison between the group of parents who did not want to participate, with the parents who did participate, was done. Finally, our paper reports only family caregivers´ perspective, pediatric patients´ insight is not included.

## Conclusion

This study brings new insight into parental and primary treating physicians’ experience with initial palliative care consultations. It highlights its positive impact on parental autonomy and parental willingness to participate in advance care planning. Our study offers several suggestions how to improve palliative practice including the presence of the primary treating physician at the consultation or giving parents the opportunity to authorize the report from the consultation. Further research should focus on how to improve understanding and expectations of palliative consultation and on exploring perspectives of pediatric patients and other health care professionals.

### Electronic supplementary material

Below is the link to the electronic supplementary material.

Supplementary Material 1

Supplementary Material 2

Supplementary Material 3

Supplementary Material 4

## Data Availability

Requests for data access should be addressed to the leading author (LH) and will be reviewed and responded to.

## Abbreviations

PPC: pediatric palliative care
LLC: life limiting condition
PPCT: pediatric palliative care team
F: family caregiver
TS: treating specialist

## References

[1] Arias-Casais N, Garralda E, Pons JJ, Marston J, Chambers L, Downing J (2020). Mapping pediatric palliative care development in the WHO-European region: children living in low-to-middle-income countries are less likely to access it. J Pain Symptom Manage.

[2] Kaye EC, Friebert S, Baker JN. Early integration of palliative care for children with high-risk cancer and their families. Vol. 63, Pediatric Blood and Cancer. 2016. p. 593–7. 10.1002/pbc.25848. 10.1002/pbc.25848 26579997

[3] Mack JW, Wolfe J (2006). Early integration of pediatric palliative care: for some children, palliative care starts at diagnosis. [Review] [20 refs]. Curr Opin Pediatr.

[4] Gans D, Kominski GF, Roby DH, Diamant AL, Chen X, Lin W et al. Better outcomes, lower costs: palliative care program reduces stress, costs of care for children with life-threatening conditions. Policy Brief UCLA Cent Health Policy Res. 2012;(PB2012-3):1–8. 22946141

[5] Groh G, Vyhnalek B, Feddersen B, Führer M, Borasio GD (2013). Effectiveness of a specialized outpatient palliative care service as experienced by patients and caregivers. J Palliat Med.

[6] Wolfe J, Hammel JF, Edwards KE, Duncan J, Comeau M, Breyer J et al. Easing of suffering in children with cancer at the end of life: is care changing ? J Clin Oncol. 2008;26(10):1717–23. 10.1200/JCO.2007.14.0277. 10.1200/JCO.2007.14.0277 18375901

[7] Sommerbakk R, Haugen DF, Tjora A, Kaasa S, Hjermstad MJ (2016). Barriers to and facilitators for implementing quality improvements in palliative care - results from a qualitative interview study in Norway. BMC Palliat Care [Internet].

[8] Davies B, Sehring SA, Partridge JC, Cooper BA, Hughes A, Philp JC (2008). Barriers to palliative care for children: perceptions of pediatric health care providers. Pediatr [Internet].

[9] Haines ER, Frost AC, Kane HL, Rokoske FS (2018). Barriers to accessing palliative care for pediatric patients with cancer: a review of the literature. Cancer.

[10] Verberne LM, Kars MC, Schepers SA, Schouten-Van Meeteren AYN, Grootenhuis MA, Van Delden JJM (2018). Barriers and facilitators to the implementation of a paediatric palliative care team. BMC Palliat Care.

[11] Benini F, Congedi S, Rusalen F, Cavicchiolo ME, Lago P. Barriers to perinatal palliative care consultation. Front Pediatr 2020;8:1–5. 10.3389/fped.2020.590616. 10.3389/fped.2020.590616 PMC7536314 33072680

[12] Walter JK, Hill DL, Didomenico C, Parikh S, Feudtner C (2019). A conceptual model of barriers and facilitators to primary clinical teams requesting pediatric palliative care consultation based upon a narrative review. BMC Palliat Care.

[13] Bergstraesser E, Hain RD, Pereira JL. The development of an instrument that can identify children with palliative care needs: the paediatric palliative screening scale (PaPaS scale): a qualitative study approach. BMC Palliat Care. 2013;12(1). 10.1186/1472-684X-12-20. 10.1186/1472-684X-12-20 PMC3663726 23657092

[14] Burke K, Coombes LH, Menezes A, Anderson A-K (2017). The ‘surprise’ question in paediatric palliative care: a prospective cohort study. Palliat Med [Internet].

[15] Kaye EC, Jerkins J, Gushue CA, DeMarsh S, Sykes A, Lu Z (2018). Predictors of late palliative care referral in children with cancer. J Pain Symptom Manage [Internet].

[16] Rapoport A, Gupta S (2021). Children and adolescents with hematologic cancers deserve better end-of-life care. Cancer [Internet].

[17] Kassam A, Sutradhar R, Widger K, Rapoport A, Pole JD, Nelson K (2017). Predictors of and trends in high-intensity end-of-life care among children with cancer: a population-based study using health services data. J Clin Oncol.

[18] Johnston DL, Vadeboncoeur C (2012). Palliative care consultation in pediatric oncology. Support Care Cancer.

[19] Moore D, Sheetz J (2014). Pediatric palliative care consultation. Pediatr Clin North Am [Internet].

[20] Bradford N, Rolfe M, Ekberg S, Mitchell G, Beane T, Ferranti K (2021). Family meetings in paediatric palliative care: an integrative review. BMJ Support Palliat Care.

[21] Daxer M, Monz A, Hein K, Heitkamp N, Knochel K, Borasio GD (2022). How to open the door: a qualitative, observational study on initiating advance care discussions with parents in pediatric palliative care. J Palliat Med.

[22] Bradford N, Herbert A, Mott C, Armfield N, Young J, Smith A (2014). Components and principles of a pediatric palliative care consultation: results of a delphi study. J Palliat Med.

[23] Verberne LM, Schouten-van Meeteren AYN, Bosman DK, Colenbrander DA, Jagt CT, Grootenhuis MA (2017). Parental experiences with a paediatric palliative care team: a qualitative study. Palliat Med.

[24] Bogetz JF, Root MC, Purser L, Torkildson C (2018). Comparing health care provider-perceived barriers to pediatric palliative care fifteen years ago and today. J Palliat Med [Internet].

[25] Clercq E, De, Rost M, Rakic M, Ansari M, Brazzola P, Wangmo T et al. The conceptual understanding of pediatric palliative care: a Swiss healthcare perspective. BMC Palliat Care. 2019;18(1):55. 10.1186/s12904-019-0438-1. 10.1186/s12904-019-0438-1 PMC6625075 31296209

[26] Todd Dalberg E, Jacob-Files PAC (2013). Pediatric oncology providers perceptions of barriers and facilitators to early integration of pediatric palliative care. Pediatr Blood Cancer.

[27] Gale NK, Heath G, Cameron E, Rashid S, Redwood S (2013). Using the framework method for the analysis of qualitative data in multi-disciplinary health research. BMC Med Res Methodol [Internet].

[28] Tong A, Sainsbury P, Craig J (2007). Consolidated criteria for reporting qualitative research (COREQ): a 32-item checklist for interviews and focus groups. Int J Qual Heal Care.

[29] Collingridge DS, Gantt EE (2008). The quality of qualitative research. Am J Med Qual [Internet].

[30] Monterosso L, Kristjanson LJ, Phillips MB (2009). The supportive and palliative care needs of Australian families of children who die from cancer. Palliat Med.

[31] Thompson LA, Knapp C, Madden V, Shenkman E. Pediatricians ’ perceptions of and preferred timing for pediatric palliative care. 2009;123(5). 10.1542/peds.2008-2721. 10.1542/peds.2008-2721 19403469

[32] Roth M, Wang D, Kim M, Moody K, Care E. An assessment of the current state of palliative care education in pediatric hematology/oncology fellowship training exposure of fellows to patients receiving. Pediatr Blood Cancer. 2009;53:647–51. 10.1002/pbc.22110. 10.1002/pbc.22110 19449397

[33] Sanderson A, Hall AM, Wolfe J (2016). Advance care discussions: pediatric clinician preparedness and practices. J Pain Symptom Manage [Internet].

[34] Krizova E, Simek J (2007). Theory and practice of informed consent in the Czech Republic. J Med Ethics.

[35] Rapoport A. Similarities and differences in Czech and canadian pediatric palliative care. 2021;2(1):49–51.

[36] Morrison W, Clark JD, Lewis-Newby M, Kon AA (2018). Titrating clinician directiveness in serious pediatric illness. Pediatrics.

[37] Zhukovsky DS, Herzog CE, Kaur G, Palmer JL, Bruera E (2009). The impact of palliative care consultation on symptom assessment, communication needs, and palliative interventions in pediatric patients with cancer. J Palliat Med.

[38] Sisk B, Baker JN. A model of interpersonal trust, credibility, and relationship maintenance. Pediatrics. 2019;144(6). 10.1542/peds.2019-1319. 10.1542/peds.2019-1319 PMC6889969 31722962

