# Supplementary material for: Innovative communication approaches for initializing pediatric palliative care: perspectives of family caregivers and treating specialists
Source: BMC Palliat Care. 2023 Oct 10;22:152. doi: 10.1186/s12904-023-01269-3 (PMC10563209; doi:10.1186/s12904-023-01269-3)
Supplement: Supplementary file 2 — Supplementary Material 2 [file 12904_2023_1269_MOESM2_ESM.docx]

| Table S2 Semistructured interview questions | |
| --- | --- |
| Questions for Family caregivers | |
| Q1 | What were your expectations before the first consultation with the pediatric palliative care team? |
| Q2 | To what extent did your original expectation correspond to the actual course and outcome of the consultation? |
| Q3 | How did you perceive the overall setting of the consultation (length, place, number of participants and their roles)? |
| Q4 | What did you perceive as most important during the initial consultation with the pediatric palliative care team? |
| Q5 | How did you perceive the possibility to provide feedback on the minutes of the consultation before it is entered into the medical documentation? |
| Questions for Primary Treating Physicians | |
| Q1 | How do you perceive the scope and content of the initial palliative consultation? |
| Q2 | How do you find the documentation of a palliative consultation different from other reports? |
| Q3 | Please try to assess the usefulness of the palliative consultation record for your decision-making and further care for the patient and their family. |
| Q4 | Parents can provide feedback on the record from the initial palliative consultation before it is saved in the documentation, which is taken into account by the palliative team in the final formulation of the record. What do you think about this process? |
