# Supplementary material for: Innovative communication approaches for initializing pediatric palliative care: perspectives of family caregivers and treating specialists
Source: BMC Palliat Care. 2023 Oct 10;22:152. doi: 10.1186/s12904-023-01269-3 (PMC10563209; doi:10.1186/s12904-023-01269-3)
Supplement: Supplementary file 4 — Supplementary Material 4 [file 12904_2023_1269_MOESM4_ESM.docx]

| Table S4 Thematic Topics |
| --- |
| 1. Expectations |
| 2. Content and Evaluation |
| 2.1 Unique Communication |
| 2.2 Practical aspects of the consultation |
| 2.3 Specific topics discussed |
| 3 Respect and support from the team |
| 3.1 Parental role |
| 3.2 Long-term support |
| 4 Consultation outcomes |
| 4.1 Written report from the initial palliative care consultation |
| 4.2 Practical information obtained |
